# Complete Genome Sequence of *Ureibacillus thermosphaericus* A1, a Thermophilic Bacillus Isolated from Compost

**DOI:** 10.1128/genomeA.00910-17

**Published:** 2017-09-21

**Authors:** Hironaga Akita, Zen-ichiro Kimura, Akinori Matsushika

**Affiliations:** a Research Institute for Sustainable Chemistry, National Institute of Advanced Industrial Science and Technology (AIST), Higashi-Hiroshima, Hiroshima, Japan; b Department of Civil and Environmental Engineering, National Institute of Technology, Kure College, Kure, Hiroshima, Japan; c Graduate School of Advanced Sciences of Matter, Hiroshima University, Higashi-Hiroshima, Hiroshima, Japan

## Abstract

*Ureibacillus thermosphaericus* A1 was isolated from compost collected in Munakata City, Fukuoka Prefecture, Japan. Here, we report the first complete genome sequence of *U. thermosphaericus*. The complete genome of this strain consists of 3,488,104 bp with a GC content of 36.3% and comprises 3,362 predicted coding sequences.

## GENOME ANNOUNCEMENT

Members of the genus *Ureibacillus* are Gram-negative thermophilic spore-forming bacteria (1). This genus was propounded from the *Bacillus thermosphaericus* cluster based on its phenotypic, chemosystematic, and phylogenetic properties (1). Six species have been reported to date. We previously isolated *Ureibacillus thermosphaericus* A1 (strain number NBRC 108682) from compost collected in Munakata City, Fukuoka Prefecture, Japan (2). This strain grows at temperatures ranging from 37°C to 55°C and exhibits rapid growth at 50°C. The bacteria produce industrially important enzymes, such as amino acid dehydrogenase (2), catalase (3), and esterase (4). Moreover, *U. thermosphaericus* was recently used as a biocatalyst for degradation of lignocellulosic biomass, which is useful for production of second-generation biofuels (5, 6). For future in-depth genomic studies and industrial applications of this bacterium, *U. thermosphaericus* A1 was subjected to genome sequencing.

A sample was prepared for genome sequencing by growing *U. thermosphaericus* A1 aerobically overnight at 50°C in nutrient broth (Kyokuto). The genomic DNA was then extracted from the cultures and purified using an Illustra bacteria genomicPrep minispin kit (GE Healthcare) according to the manufacturer’s instructions. The concentration and purity of the genomic DNA were measured using a NanoDrop ND-1000 spectrophotometer (Thermo Fisher Scientific) and a Quant-iT double-stranded DNA (dsDNA) broad range (BR) assay kit (Invitrogen). After fragmentation of the genomic DNA (31.5 µg) into pieces of approximately 20 kb using g-TUBE (Covaris), the resulting fragments were ligated to SMRTbell sequencing adapters using a SMRTbell template prep kit 1.0 (Pacific Biosciences), yielding SMRTbell libraries. The library size was measured using Agilent 2200 TapeStation (Agilent Technologies). The SMRTbell libraries were then bound to polymerases and sequencing primers using a DNA/polymerase binding kit P6 version 2 (Pacific Biosciences), yielding the sequencing templates. The concentrations of the sequencing templates were calculated using Binding Calculator version 2.3.1.1 (Pacific Biosciences), after which the templates were bound to MagBeads using a MagBead kit (Pacific Biosciences) and loaded onto SMRT Cells 8Pac version 3 (Pacific Biosciences). Sequencing was then performed using PacBio RS II (Pacific Biosciences).

The raw data included 99,029 reads with 407× coverage and were assembled *de novo* using SMRT Analysis version 2.3.0 (Pacific Biosciences) (7) to filter the subreads. The genome sequence was 3,488,104 bp, and the GC content was 36.3%. Genome annotation was performed using CRITICA (8) and Glimmer2 (9), and 3,362 predicted coding sequences were identified. In addition, 83 tRNA genes and 18 rRNA genes were detected using tRNAScan-SE (10) and BLASTN (11), respectively.

### Accession number(s).

The complete genome sequence of *U. thermosphaericus* A1 has been deposited in DDBJ/EMBL/GenBank under the accession number AP018335.

## References

[1] Fortina MG, Pukall R, Schumann P, Mora D, Parini C, Manachini PL, Stackebrandt E 2001 *Ureibacillus* gen. nov., a new genus to accommodate *Bacillus thermosphaericus* (Andersson et al. 1995), emendation of *Ureibacillus thermosphaericus* and description of *Ureibacillus terrenus* sp. nov. Int J Syst Evol Microbiol 51:447–455. doi:10.1099/00207713-51-2-447. 11321090

[2] Akita H, Fujino Y, Doi K, Ohshima T 2011 Highly stable *meso*-diaminopimelate dehydrogenase from an *Ureibacillus thermosphaericus* strain A1 isolated from a Japanese compost: purification, characterization and sequencing. AMB Express 1:43. doi:10.1186/2191-0855-1-43. 22117688 PMC3293039

[3] Jia X, Lin X, Tian Y, Chen J, You M 2017 High production, purification, biochemical characterization and gene analysis of a novel catalase from the thermophilic bacterium *Ureibacillus thermosphaericus* FZSF03. Int J Biol Macromol 103:89–98. doi:10.1016/j.ijbiomac.2017.05.034. 28501604

[4] Gagné A, Chicoine M, Morin A, Houde A 2001 Phenotypic and genotypic characterization of esterase-producing *Ureibacillus thermosphaericus* isolated from an aerobic digestor of swine waste. Can J Microbiol 47:908–915. doi:10.1139/w01-096. 11718544

[5] Zainudin MH, Hassan MA, Tokura M, Shirai Y 2013 Indigenous cellulolytic and hemicellulolytic bacteria enhanced rapid co-composting of lignocellulose oil palm empty fruit bunch with palm oil mill effluent anaerobic sludge. Bioresour Technol 147:632–635. doi:10.1016/j.biortech.2013.08.061. 24012093

[6] Asada C, Sasaki C, Takamatsu T, Nakamura Y 2015 Conversion of steam-exploded cedar into ethanol using simultaneous saccharification, fermentation and detoxification process. Bioresour Technol 176:203–209. doi:10.1016/j.biortech.2014.11.039. 25461004

[7] Chin CS, Alexander DH, Marks P, Klammer AA, Drake J, Heiner C, Clum A, Copeland A, Huddleston J, Eichler EE, Turner SW, Korlach J 2013 Nonhybrid, finished microbial genome assemblies from long-read SMRT sequencing data. Nat Methods 10:563–569. doi:10.1038/nmeth.2474. 23644548

[8] Badger JH, Olsen GJ 1999 CRITICA: coding region identification tool invoking comparative analysis. Mol Biol Evol 16:512–524. doi:10.1093/oxfordjournals.molbev.a026133. 10331277

[9] Delcher AL, Harmon D, Kasif S, White O, Salzberg SL 1999 Improved microbial gene identification with Glimmer. Nucleic Acids Res 27:4636–4641. doi:10.1093/nar/27.23.4636. 10556321 PMC148753

[10] Lowe TM, Eddy SR 1997 tRNAscan-SE: a program for improved detection of transfer RNA genes in genomic sequence. Nucleic Acids Res 25:955–964. 9023104 10.1093/nar/25.5.955 PMC146525

[11] Altschul SF, Gish W, Miller W, Myers EW, Lipman DJ 1990 Basic local alignment search tool. J Mol Biol 215:403–410. doi:10.1016/S0022-2836(05)80360-2. 2231712

